# MicroRNAs in adipose tissue fibrosis: Mechanisms and therapeutic potential

**DOI:** 10.1016/j.gendis.2024.101287

**Published:** 2024-04-05

**Authors:** Mei Tian, Yang Zhou, Yitong Guo, Qing Xia, Zehua Wang, Xinying Zheng, Jinze Shen, Junping Guo, Shiwei Duan, Lijun Wang

**Affiliations:** a College of Pharmacy, Zhejiang University of Technology, Hangzhou, Zhejiang 310014, China; b Geriatric Medicine Center, Department of Endocrinology, Zhejiang Provincial People's Hospital (Affiliated People's Hospital), Hangzhou Medical College, Hangzhou, Zhejiang 310014, China; c Key Laboratory of Novel Targets and Drug Study for Neural Repair of Zhejiang Province, School of Medicine, Hangzhou City University, Hangzhou, Zhejiang 310015, China; d Rainbowfish Rehabilitation and Nursing School, Hangzhou Vocational & Technical College, Hangzhou, Zhejiang 310018, China; e Department of Clinical Medicine, School of Medicine, Hangzhou City University, Hangzhou, Zhejiang 310015, China

**Keywords:** Adipose tissue fibrosis, Adipose-derived stem cell, Extracellular matrix, miRNAs, Signaling pathways

## Abstract

Adipose tissue fibrosis, characterized by abnormal extracellular matrix deposition within adipose tissue, signifies a crucial indicator of adipose tissue malfunction, potentially leading to organ tissue dysfunction. Various factors, including a high-fat diet, non-alcoholic fatty liver disease, and insulin resistance, coincide with adipose tissue fibrosis. MicroRNAs (miRNAs) represent a class of small non-coding RNAs with significant influence on tissue fibrosis through diverse signaling pathways. For instance, in response to a high-fat diet, miRNAs can modulate signaling pathways such as TGF-β/Smad, PI3K/AKT, and PPAR-γ to impact adipose tissue fibrosis. Furthermore, miRNAs play roles in inhibiting fibrosis in different contexts: suppressing corneal fibrosis via the TGF-β/Smad pathway, mitigating cardiac fibrosis through the VEGF signaling pathway, reducing wound fibrosis via regulation of the MAPK signaling pathway, and diminishing fibrosis post-fat transplantation via involvement in the PDGFR-β signaling pathway. Notably, the secretome released by miRNA-transfected adipose-derived stem cells facilitates targeted delivery of miRNAs to evade host immune rejection, enhancing their anti-fibrotic efficacy. Hence, this study endeavors to elucidate the role and mechanism of miRNAs in adipose tissue fibrosis and explore the mechanisms and advantages of the secretome released by miRNA-transfected adipose-derived stem cells in combating fibrotic diseases.

## Introduction

Adipose tissue plays a vital role in the human body, but prolonged damage or inflammation can lead to abnormal fibrous tissue proliferation, resulting in adipose tissue fibrosis.^1^, ^2^, ^3^ This condition, characterized by the abnormal deposition of extracellular matrix (ECM), primarily involves collagen cross-linking and myofibroblast activation.^4^ Adipose tissue fibrosis is a significant indicator of adipose tissue dysfunction, and excessive fibrosis can impair organ tissue function.^5^ Various cells within adipose tissue, including adipocytes, adipose-derived stem cells (ADSCs), endothelial cells, and fibroblasts, collectively regulate the fibrosis process.^6^^,^^7^ Factors such as high-fat diet, non-alcoholic fatty liver disease, and insulin resistance are associated with adipose tissue fibrosis.^5^^,^^8^^,^^9^

MicroRNAs (miRNAs), a class of non-coding RNAs with lengths ranging from 20 to 24 nucleotides, regulate gene expression through transcriptional degradation or translational inhibition.^10^ Mature miRNAs are integrated into the RNA-induced silencing complex, recognizing target mRNA through partially complementary base pairing, leading to mRNA translation inhibition or destabilization.^11^, ^12^, ^13^ miRNAs play crucial roles in regulating inflammation and metabolic dysfunction processes linked to obesity,^14^^,^^15^ impacting cytokine and adipokine levels.^16^^,^^17^ Consequently, they influence the course and outcome of adipose tissue fibrosis. Notably, studies reveal the potential of miRNA-transfected ADSCs (miRNA-ADSCs), whose secretions can deliver miRNA to target sites, avoiding immune rejection and enhancing anti-fibrotic effects.^18^, ^19^, ^20^, ^21^ Moreover, these secretions can directly act on target sites, exerting anti-fibrotic effects.^22^

Adipose tissue fibrosis is a prevalent metabolic disorder closely associated with chronic conditions like obesity, diabetes, and cardiovascular disease.^23^ miRNAs, as essential gene expression regulators, are intricately involved in the onset and progression of adipose tissue fibrosis.^24^ miRNAs have been found to bind to target genes within various signaling pathways, including TGF-β/Smad, PI3K/AKT, and PPAR-γ, thereby inhibiting signal transduction and suppressing the expression of fibrotic genes, ultimately leading to an anti-fibrotic effect. Moreover, emerging research suggests that miRNA-engineered ADSCs, referred to as miRNA-ADSCs, can be generated by transfection of miRNA into ADSCs.^24^^,^^25^ These engineered cells secrete miRNA-containing vesicles, facilitating targeted delivery to specific sites, thus minimizing the risk of immune rejection and enhancing the effectiveness of the anti-fibrotic response.^18^, ^19^, ^20^, ^21^ Furthermore, these secreted miRNA vesicles can directly target fibrotic sites, exerting their anti-fibrotic effects.^22^

This comprehensive review systematically explores the functions and mechanisms of miRNAs in adipose tissue fibrosis, encompassing the signaling pathways they participate in, the gene and protein targets they regulate, and incorporating the latest experimental and clinical research findings. By delving into the potential of miRNAs as diagnostic biomarkers and therapeutic targets for adipose tissue fibrosis, this review aims to serve as a valuable reference, fostering a deeper understanding of the molecular intricacies of adipose tissue fibrosis and facilitating the quest for effective diagnostic and therapeutic interventions.

### miRNAs in non-alcoholic fatty liver disease and non-alcoholic steatohepatitis: insights into fibrosis development

Non-alcoholic fatty liver disease (NAFLD) stands as a prevalent liver disorder associated with obesity, affecting around 25% of the global population.^26^ It is characterized by the accumulation of fat content in the liver exceeding 5% in the absence of apparent alcohol consumption or other liver diseases.^27^ Steatosis, a hallmark of NAFLD, occurs when the liver fails to metabolize surplus free fatty acids, leading to the accumulation of triglycerides within liver cells. Notably, a subset of NAFLD patients may progress to non-alcoholic steatohepatitis (NASH), a condition significantly predisposing individuals to liver fibrosis.^28^ The progression from NAFLD to NASH might be linked to alterations in the levels of soluble signaling molecules, including cytokines and adipokines secreted by adipose tissue.^17^ miRNAs play a pivotal role in the development of both NAFLD and NASH. Estep et al conducted an extensive study, analyzing a wide array of human miRNAs. Their findings revealed significant differential expression of 113 miRNA types in the visceral adipose tissue of NASH patients. Among these, 35 miRNAs, such as hsa-miR-197, hsa-miR-146b-3p, and hsa-miR-149, exhibited distinct expression patterns in the visceral adipose tissue of NASH patients with fibrosis compared with non-NASH patients.^16^ These specific miRNAs may influence the interplay between visceral adipose tissue and the liver by regulating signaling pathways, genes, or protein targets, ultimately contributing to the onset of liver fibrosis.^16^

## TGF-β/smad signaling and miRNAs: decoding their roles in liver fibrosis progression

**Fig. 1:**
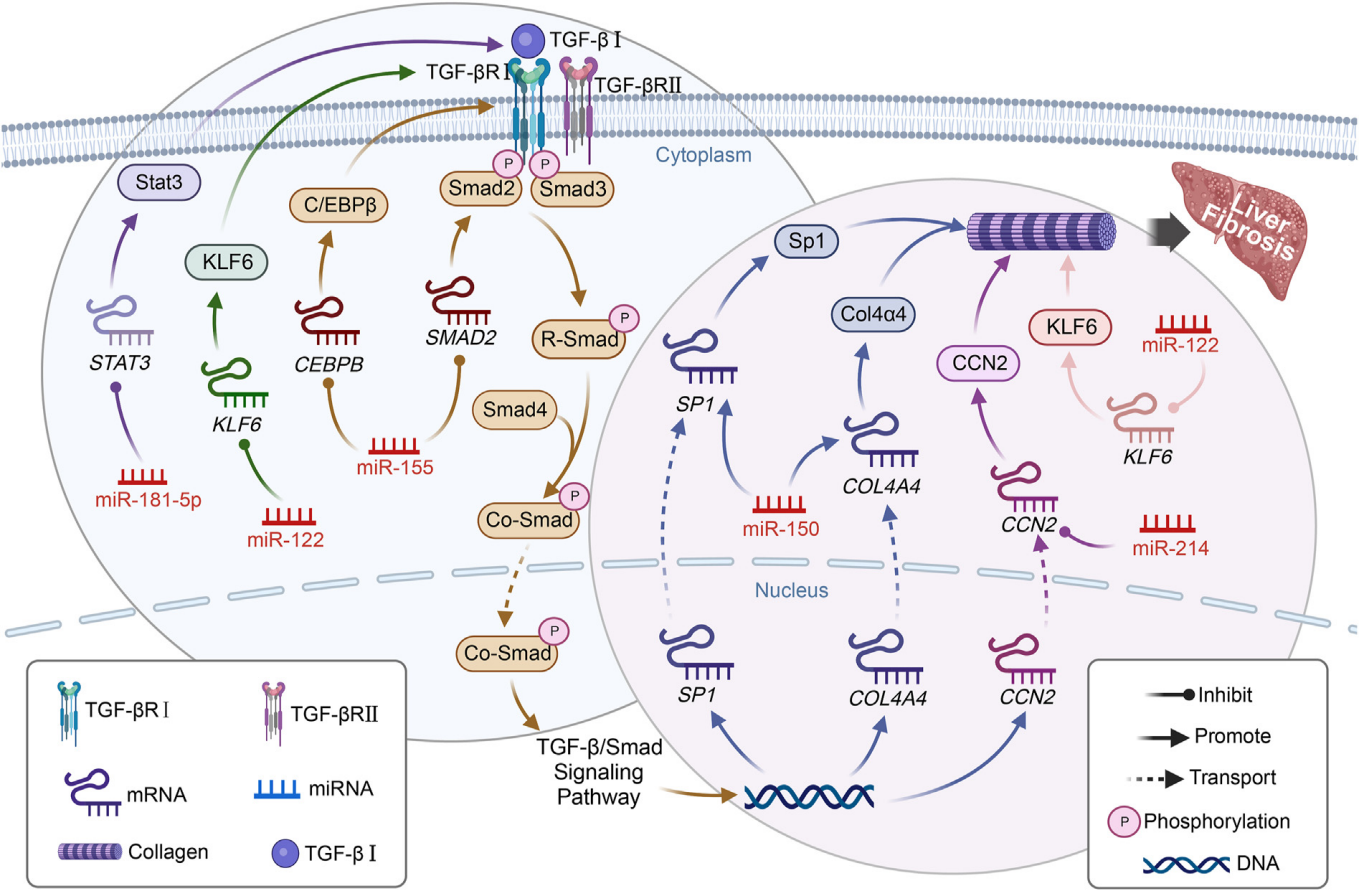
TGF-β/Smad signaling pathway and miRNA regulation in liver fibrosis. Tissue fibrosis intricately links with the TGF-β/Smad signaling pathway. Transforming growth factor βⅠ(TGF-βⅠ) binds with the TGF-βⅠreceptor (TGF-βRⅠ) on hepatic stellate cells, forming a complex. This complex phosphorylates R-Smad, created by Smad2 and Smad3 pairing. R-Smad combines with Smad4, forming the Co-Smad complex (a complex with Smad2, Smad3, and Smad4), which translocates from the cytoplasm to the nucleus, regulating collagen expression, a key fibrotic protein. Various miRNAs impact liver fibrosis progression through TGF-β/Smad signaling, including miR-122, miR-155, miR-181-5p, miR-150, and miRNA-214. miR-122 targets Kruppel-like factor 6 (KLF6), inhibits TGF-βRⅠ activation, dampens TGF-β/Smad signaling, and hinders liver fibrosis. miR-155 binds to *SMAD2*, elevates Smad2 levels, and intensifies liver fibrosis induced by TGF-β/Smad signaling. It also targets *CEBPB*, hindering the activation of C/EBPβ (CCAAT/enhancer-binding protein beta), promoting TGF-βⅠ activation, and aggravating liver fibrosis. miR-181-5p down-regulates Stat3 (signal transducer and activator of transcription 3) by targeting *STAT3*, inhibits TGF-βⅠ activation, and attenuates TGF-β/Smad signaling. miR-150 directly targets *SP1* and *COL4A4*, reducing Sp1 (specificity protein 1) and Col4α4 (collagen type IV alpha 4 chain) expression, lowering collagen levels, and inhibiting liver fibrosis. miR-214 lowers CCN2 (cellular communication network factor 2) expression by binding to *CCN2* under the TGF-β/Smad signaling pathway, diminishing collagen synthesis, and effectively inhibiting liver fibrosis. Smad, mothers against decapentaplegic homolog.

TGF-β/Smad signaling represents a crucial pathway closely associated with fibrosis in various tissues. This pathway accelerates liver fibrosis by promoting hepatic stellate cell (HSC) activation, stimulating collagen gene transcription, or inhibiting the expression of matrix metalloproteinases.^29^^,^^30^ Specifically, transforming growth factor-βⅠ (TGF-βⅠ) binds to the TGF-βⅠ receptor (TGF-βRⅠ) on HSCs, initiating a complex phosphorylation cascade involving R-Smad, Smad4, and Co-Smad (a complex with Smad2, Smad3, and Smad4), which subsequently regulates the expression of fibrosis-related genes and influences ECM composition.^31^, ^32^, ^33^, ^34^ As shown in Table 1 and Figure 1, Numerous miRNAs modulate liver fibrosis progression through the TGF-β/Smad signaling pathway, such as miR-122, miR-150, miR-155, miR-181-5p, and miR-214. These miRNAs either directly target key molecules within the pathway, like TGF-βR and Smad proteins, or indirectly impact other signaling pathways to inhibit or promote liver fibrosis.

**Table 1:** Targets and roles of miRNAs in adipose tissue fibrosis.

Signaling pathways	miRNA	Targets	Binding site of miRNA and mRNA		Role in fibrosis	Reference
			Binding site of miRNA (3′- … -5′)	Binding site of mRNA (5′- … -3′)		
*Fibrosis of liver tissue*						
TGF-β/Smad	miR-122	Col1α1 (*KLF6*)	—	—	Inhibit	^35^
	miR-150	Sp1 (*SP1*)	*AACCCUCU*	*UUGGGAGA*	Inhibit	^43^
		Col4α4 (*COL4A4*)	*AACCCUCU*	*UUGGGAGA*	Inhibit	^43^
	miR-155	C/EBPβ (*CEBPB*)	*GGG--auAGUgcuaaUCGUAAUU*	*UCCguuUCA-------AGCAUUAA*	Promote	^37^
		Smad2 (SMAD2)	*CGUAAUU-----uaaUCGUAAUU*	*GCAUUAAc---ccuAGCAUUAA*	Promote	^38^
	miR-181-5p	Bcl-2 (*BCL2*)	*CUUACA*	*GAAUGU*	Inhibit	^25^
		*Stat3 (STAT3)*	*ACUUAC*	*UGAAUG*	Inhibit	^25^
	miR-214	CCN2 (*CCN2*)	*UgacGGa-caGAcaCGGA-C--ga-cA*	*UauagcuGaUCAGuuUUuucaCCuggA*	Inhibit	^24^
HIF-1α/LOX	miR-122	HIF-1α (*HIF1A*)	*UGUgGUaacAGUGU-GAGG*	*ACAgCAcauUCACAgCUAA*	Inhibit	^48^
MAP3K3	miR-122	MAP3K3 (*MAP3K3*)	*GUGAGG*	*CACUCC*	Inhibit	^48^
NF-κB	miR-150	C-Myb (*MYB*)	*UGUGGUAACAGUGU-GAGG*	*UCACAGCACAUUCACAGCUCC*	Inhibit	^43^
P4Hα1/Col1α1	miR-122	P4Hα1 (*P4HA1*)	–	–	Inhibit	^54^
*High-fat diet-induced fibrosis*						
TGF-β/Smad	miR-140	Smad3 (*SMAD3*)	–	–	Inhibit	^64^
	miR-410-5p	Smad7 (*SMAD7*)	*GggCU---gCcGgGACAgCC*	*GggCU---gCcGgGACAgCC*	Promote	^65^
PI3K/AKT	miR-30b	Runx1 (*RUNX1*)	*ACAAAUG*	*UGUUUAC*	Inhibit	^73^
PPAR-γ	miR-155	PPAR-γ (*PPARG*)	*AUagugC-UaaUCGUAA*	*UAcuguGaAaaAGCAUU*	Promote	^76^
*Other types of fibrosis*						
TGF-β/Smad	miR-19a	HIPK2 (*HIPK2*)	*AgucaaAacguaucuaAACGUG*	*UauuccUcaauguaauUGCACA*	Inhibit	^22^
VEGF	miR-126	Spred1 (*SPRED1*)	*—*	*—*	Inhibit	^81^
		PIK3R2 (*PIK3R2*)	*—*	*—*		
MAPK	miR-146b-5p	PDGFR-α (*PDGFRA*)	*CGgauAC-CuUaAGUCAagAGT*	*GCucuUGaGgAgUCAGUguUCA*	Inhibit	^85^
PDGFR-β	miR-24-3p	PDGFR-β (*PDGFRB*)	—	—	Inhibit	^94^

Notes: Transcription factors are indicated in italics. AKT, protein kinase B; Bcl-2, B-cell CLL/lymphoma 2; CCN2, cellular communication network factor 2; C-myb, MYB proto-oncogene, transcription factor; Col1α1, collagen type I alpha 1 chain; COL4A4, collagen type IV alpha 4 chain; C/EBPβ, CCAAT enhancer binding protein beta; HIF-1α, hypoxia inducible factor 1 subunit alpha; HIPK2, homeodomain interacting protein kinase 2; KLF6, Kruppel-like factor 6; LOX, lysyl oxidase; MAP3K3, mitogen-activated protein kinase kinase kinase 3; MAPK, mitogen-activated protein kinase; NF-κB, nuclear factor kappa-light-chain-enhancer of activated B cells; P4Hα1, prolyl 4-hydroxylase subunit alpha 1; PDGFR-α, platelet derived growth factor receptor alpha; PDGFR-β, platelet derived growth factor receptor beta; PI3K, phosphoinositide 3 kinase; PIK3R2, phosphoinositide-3-kinase regulatory subunit 2; PPAR-γ, peroxisome proliferator activated receptor gamma; Runx1, Runt related transcription factor 1; Smad, mothers against decapentaplegic homolog; Smad2, SMAD family member 2; Smad3, SMAD family member 3; Smad7, SMAD family member 7; SP1, specificity protein 1; SPRED1, Sprouty related EVH1 domain containing 1; Stat3, signal transducer and activator of transcription 3; TGF-β, transforming growth factor beta; VEGF, vascular endothelial growth factor.

miR-122, a highly expressed miRNA in the liver, plays a significant role in lipid metabolism and fatty liver disease. In fibrotic liver tissues, miR-122 expression is notably down-regulated. miR-122 overexpression inhibits the translation of Krüppel-like factor 6 by delivering its secretome to HSCs, ultimately reducing collagen synthesis and inhibiting liver fibrosis.^35^^,^^36^

miR-155, a key miRNA involved in inflammation, immune responses, and fibrosis, is up-regulated in liver fibrosis. By targeting molecules like Smad2 and C/EBPβ^37^ within the TGF-β/Smad pathway, miR-155 promotes HSC activation and ECM secretion, exacerbating liver fibrosis progression.^38^^,^^39^

miR-181–5p, vital in immune and inflammatory responses, suppresses HSC survival and activation by targeting *STAT3* (signal transducer and activator of transcription) 3. This inhibition alleviates liver fibrosis by disrupting TGF-βⅠ signaling and reducing fibrotic factor expression.^25^^,^^40^

miR-150, an anti-fibrosis miRNA, inhibits HSC activation and liver fibrosis progression. In miR-150-overexpressing ADSCs, the secretome delivers anti-fibrotic miR-150 to HSCs, targeting *SP1* (specificity protein 1) and *Col4A4*. This reduces collagen synthesis, inhibiting liver fibrosis.^41^, ^42^, ^43^

miR-214, a versatile miRNA in various biological processes and fibrosis, attenuates liver fibrosis by targeting connective tissue growth factor (CCN2). The down-regulation of CCN2 expression inhibits ECM secretion, slowing liver fibrosis progression.^24^^,^^44^

## miR-122 modulates HIF-1α/LOX, MAPK, and P4Hα1/Col1α1 signaling pathways: implications for liver fibrosis regulation

**Fig. 2:**
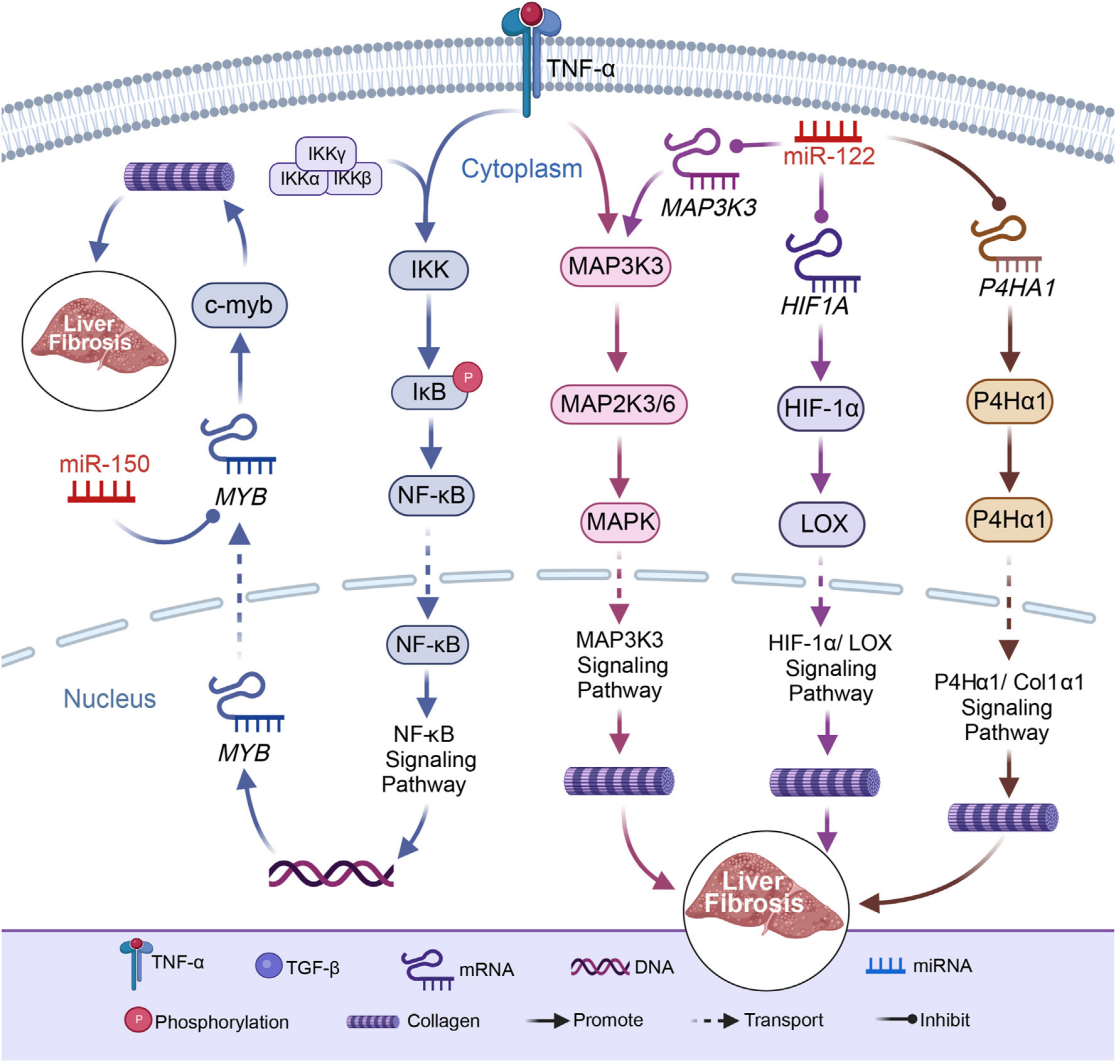
Decoding miRNA-mediated regulation of fibrotic pathways: Insights into liver fibrosis inhibition. Hypoxia-inducible factor 1-alpha (HIF-1α) enhances collagen and elastin expression by targeting LOX (lysyl oxidase), promoting liver fibrosis. miR-122 binds to *HIF1A*, inhibiting HIF-1α expression, preventing HIF-1α from activating the LOX pathway. This inhibition curtails collagen synthesis, effectively mitigating liver fibrosis. Tumor necrosis factor-α (TNF-α) triggers MAP3K3 (mitogen-activated protein kinase kinase 3) activation, leading to phosphorylation of downstream molecule MAP2K3/6 (mitogen-activated protein kinase kinase 3/6). Phosphorylated MAP2K3/6 activates subsequent MAPK molecules, orchestrating the transcription of fibrotic genes and regulating fibrotic responses. miR-122 targets MAP3K3, suppressing its expression, disrupting the MAPK pathway, reducing collagen synthesis, and attenuating fibrosis in steatohepatitis. TNF-α activates the surface receptor's IKK complex, phosphorylating and degrading IκB (inhibitor of NF-κB). With IκB degraded, activated NF-κB translocates to the nucleus, regulating gene transcription, including *MYB*. miR-150 inhibits collagen synthesis by targeting *MYB* and suppressing C-myb (MYB proto-oncogene) expression, consequently impeding liver fibrosis. Prolyl 4-hydroxylase subunit alpha 1 (P4Hα1) facilitates Col1α1 (collagen type I alpha 1 chain) folding and stabilization by catalyzing proline residues' hydroxylation on the collagen α chain, influencing collagen I synthesis. miR-122 binds to its target gene *P4HA1*, inhibiting P4Hα1 expression, thereby diminishing liver tissue collagen levels and hindering liver fibrosis progression. MAPK, mitogen-activated protein kinase; NF-κB, nuclear factor kappa-light-chain-enhancer of activated B cells.

The HIF-1α/LOX signaling pathway plays a crucial role in the progression of steatohepatitis and fibrosis. As shown in Table 1 and Figure 2, under hypoxic conditions, HIF-1α enhances collagen and elastin expression by targeting lysyl oxidase (LOX), thereby increasing the stiffness and stability of ECM and promoting liver fibrosis.^45^ Notably, LOX expression significantly rises in mice with steatohepatitis and fibrosis.^46^ In this context, miR-122 assumes a vital role and exhibits a reverse regulatory effect in HSCs. Abundantly expressed in HSCs and fibroblasts, miR-122 exerts anti-fibrotic effects.^36^^,^^47^ Specifically, miR-122 targets the gene *HIF1A*, inhibiting HIF-1α (hypoxia-inducible factor 1-alpha) translation and preventing HIF-1α from activating the LOX signaling pathway.^48^ Consequently, miR-122 stimulates collagen synthesis, reduces ECM deposition, and mitigates liver stiffness and fibrosis.

Additionally, the MAPK signaling pathway emerges as a pivotal player in liver fibrosis, orchestrating fibroblast proliferation, migration, and ECM component secretion, including collagen. As shown in Figure 1, external stimuli like bacterial infections or the inflammatory factor tumor necrosis factor-α activate mitogen-activated protein kinase MAP3K3 (also known as MEKK3), initiating downstream phosphorylation of mitogen-activated protein kinase 3/6 (MAP2K3/6). This activation cascades to mitogen-activated protein kinase (MAPK) molecules. MAPK is subsequently translocated from the cytoplasm to the nucleus, where it triggers the activation of fibrosis-related genes such as matrix metalloproteinase genes and collagen genes. This up-regulates collagen expression, thus orchestrating the fibrotic response.^2^

On the other hand, miR-122 exerts inhibitory effects on MAP3K3 translation by directly targeting it.^48^^,^^49^ Consequently, this inhibition impedes the activation of downstream signaling molecules such as MAP2K3/6, thereby interrupting the MAPK signaling pathway and suppressing the expression of fibrosis-associated factors such as matrix metalloproteinase 1 and type I collagen α chain (Col1α1). Ultimately, this cascade of events leads to diminished collagen synthesis and secretion, alleviating the cross-linking and stiffening of the ECM, and ultimately mitigating fibrosis in steatohepatitis.^48^^,^^50^

The P4Hα1/Col1α1 signaling pathway, crucial for ECM regulation, involves the interaction between prolyl-4-hydroxylase α1 (P4Hα1) and Col1α1. P4Hα1, a hydroxylase, adds a hydroxyl group to proline residues in the collagen alpha chain, converting it into hydroxyproline. This reaction promotes correct folding and stabilization of Col1α1, enhancing ECM cross-linking and stiffening ability.^51^, ^52^, ^53^ Central to HSC activation and ECM synthesis, the P4Hα1/Col1α1 pathway serves as a vital molecular marker and therapeutic target for liver fibrosis. As shown in Table 1 and Figure 2, Lou et al^54^ discovered that in ADSCs overexpressing miR-122, the miR-122 secretome can effectively inhibit liver fibrosis progression in HSCs. Their research demonstrated that miR-122 operates by reducing P4Hα1 expression through binding to its target gene *P4HA1*. Given P4Hα1's role as an upstream regulator of Col1α1, its down-regulation leads to reduced Col1α1 levels.^51^^,^^52^ Consequently, collagen content in the ECM decreases, mitigating the severity of liver fibrosis.^53^

## miR-150 secretome: a potential therapeutic strategy targeting NF-κB mediated liver fibrosis

Nuclear factor kappa-light-chain-enhancer of activated B cells (NF-κB), a crucial transcription factor in the immune system, orchestrates various cellular processes such as inflammation, immunity, and apoptosis through the regulation of multiple genes.^55^ As shown in Figure 2, its activation involves complex molecular interactions and signaling branches, with cytokine receptor-mediated signaling being a common NF-κB activation pathway. For instance, cytokines like tumor necrosis factor-α and interleukin 1 beta activate the IKK complex on their surface receptors, leading to the phosphorylation and degradation of inhibitors of NF-κB.^56^ Upon the degradation of inhibitors of NF-κB, activated NF-κB translocates to the nucleus, regulating the transcription and translation of specific genes, including *MYB*, thus influencing C-myb (MYB proto-oncogene) expression.^57^ C-myb, a transcription factor involved in cell cycle regulation and differentiation, promotes liver fibrosis by enhancing fibroblast proliferation and collagen secretion.^57^

Paik et al^43^ demonstrated that the secretome of miR-150 in ADSCs overexpressing miR-150 can be delivered to HSCs. As shown in Table 1, by targeting *MYB* and inhibiting C-myb expression, this secretome suppresses NF-κB pathway activation and reduces the synthesis of type I collagen and alpha-smooth muscle actin, effectively impeding liver fibrosis progression. Given the down-regulation of miR-150 in fibrotic liver tissue, the miR-150 secretome delivered to HSCs through miR-150-expressing ADSCs exerts potent anti-fibrotic effects.^43^

### Decoding the role of miRNAs in obesity-related adipose tissue fibrosis: insights for prevention and treatment strategies

Obesity stands as a significant contributor to adipose tissue fibrosis, characterized by the excessive accumulation of lipids in adipocytes, leading to hypoxia and inflammatory responses within the adipose tissue.^9^ These responses drive adipocyte proliferation and hypertrophy, alongside fibroblast activation.^58^ Upon activation, fibroblasts secrete substantial amounts of ECM.^59^^,^^60^ Excessive ECM accumulation results in adipose tissue hardening and rigidification, disrupting its normal physiological functions and metabolic regulation.

miRNAs, small RNA molecules regulating gene expression, play a pivotal role in obesity-related adipose tissue fibrosis. By binding to the 3′ untranslated region, miRNAs can either inhibit or promote the translation of target genes.^61^ miRNAs play a pivotal role in the development of obesity-related adipose tissue fibrosis. They exert their influence by modulating a diverse array of fibrosis-related genes and signaling pathways, including but not limited to TGF-β, Smad, and PI3K.^44^ In doing so, miRNAs can either promote or inhibit the progression of adipose tissue fibrosis, thereby significantly impacting metabolic health. Investigating the mechanisms of miRNA involvement in adipose tissue fibrosis not only enhances our understanding of the condition but also provides valuable insights and potential targets for preventing and treating obesity-related metabolic disorders and associated diseases.

## Regulatory role of miRNAs in obesity-induced adipose tissue fibrosis

**Fig. 3:**
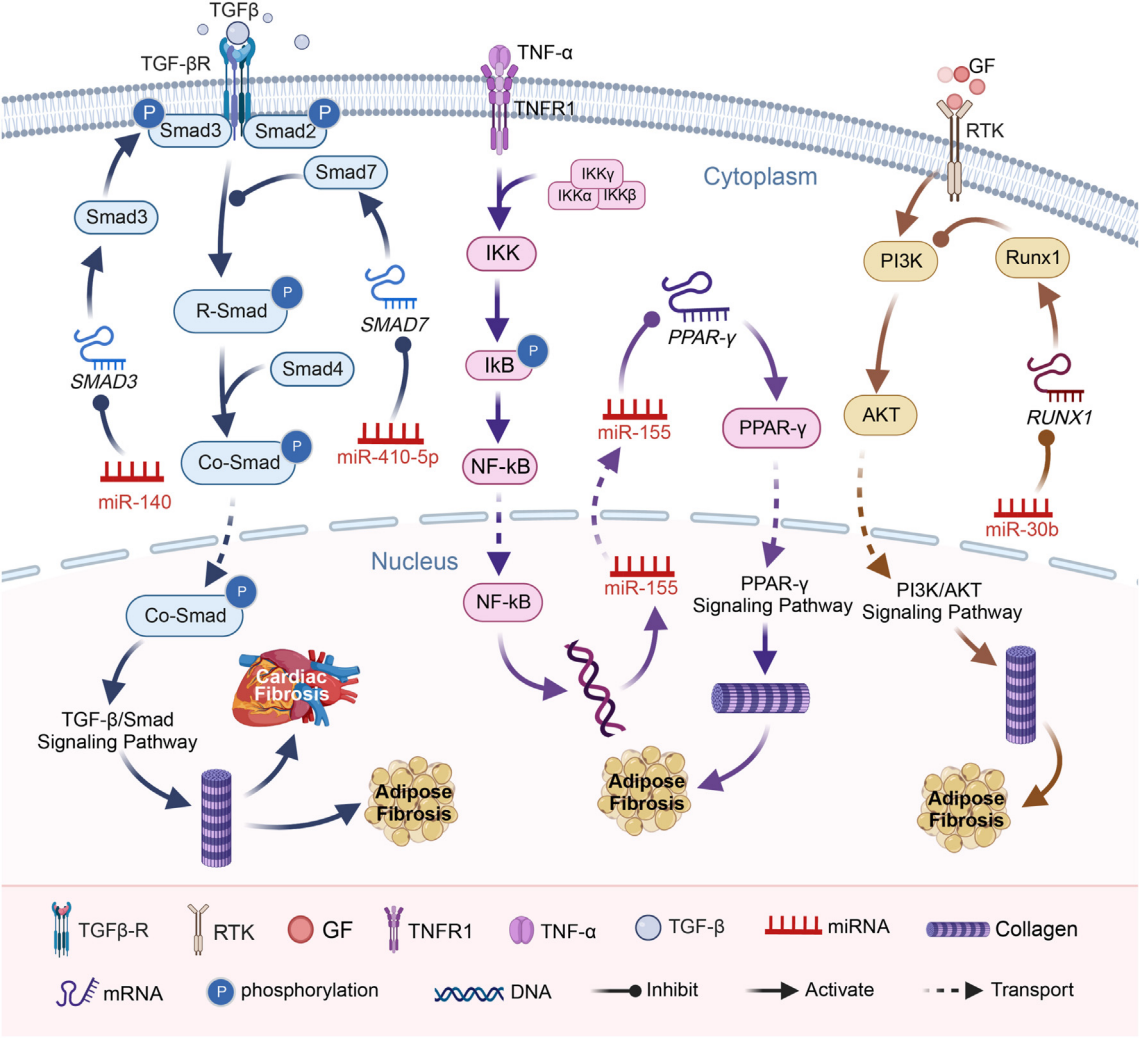
The role and therapeutic potential of miRNAs in adipose tissue fibrosis induced by high-fat diet. Transforming growth factor β (TGF-β) activates Smad3 upon binding to its receptor, and miR-140 interferes with phosphorylated *SMAD3*, inhibiting Smad3 expression. Consequently, this inhibition disrupts the TGF-β/Smad signaling pathway, decreasing fibrotic protein levels and impeding adipose tissue fibrosis. Conversely, miR-410-5p targets *SMAD7*, activating the TGF-β/Smad pathway and promoting adipose tissue fibrosis by enhancing Smad7 expression. Growth factors (GFs) activate PI3K (phosphoinositide 3 kinase) via receptor tyrosine kinase (RTK) receptors, triggering AKT (protein kinase B) phosphorylation. AKT, when activated, influences collagen synthesis. miR-30b targets *RUNX1*, reducing Runx1 (Runt related transcription factor 1) expression, which subsequently down-regulates PI3K and inhibits AKT activation. This regulatory mechanism effectively hinders adipose tissue fibrosis. Tumor necrosis factor-α (TNF-α) activates the NF-κB signaling system, elevating miR-155 expression. miR-155 targets *PPARG*, reducing PPAR-γ (peroxisome proliferator-activated receptor gamma) expression and inhibiting collagen production, effectively mitigating adipose tissue fibrosis. NF-κB, nuclear factor kappa-light-chain-enhancer of activated B cells; Smad, mothers against decapentaplegic homolog.

miR-140, a small RNA abundantly expressed in preadipocytes, plays a vital role in normal ADSC adipogenesis.^62^^,^^63^ However, in a high-fat diet-induced obesity mouse model, decreased miR-140 expression in mammary adipose tissue activates the TGF-β/Smad signaling pathway, leading to myofibroblast differentiation and ECM synthesis, ultimately causing adipose tissue fibrosis,^58^ as shown in Table 1 and Figure 3. High-fat diet-induced elevation in TGF-β levels disrupts the intricate balance between miR-140, Smad3, and TGF-β. This disruption amplifies myofibroblast differentiation and ECM synthesis, promoting adipose tissue fibrosis. Wang et al^64^ demonstrated that restoring miR-140 expression inhibits the TGF-β/Smad pathway, preventing epithelial–mesenchymal transition and improving adipose tissue fibrosis.

miR-410–5p, specifically expressed in kidney and adipose tissue, is up-regulated in a high-fat diet-induced obesity mouse model.^65^ As shown in Table 1 and Figure 3, by targeting SMAD7 and inhibiting its expression, miR-410–5p promotes the activation of the TGF-β signaling pathway.^65^^,^^66^ Zou et al^65^ discovered that miR-410–5p from kidney or adipose tissue can reach the heart through circulation, reducing Smad7 levels in cardiac tissue. Reduced Smad7 levels activate the TGF-β/Smad pathway, up-regulating fibrosis-related genes like *CCN2*, *COL1A1*, and *COL4A4*. This process significantly increases collagen production, ultimately promoting cardiac fibrosis.

Understanding the intricate regulatory networks involving miR-140 and miR-410-5p provides valuable insights into the molecular mechanisms underlying obesity-induced adipose tissue and cardiac fibrosis.

## Fine-tuning adipose tissue fibrosis: the miR-30b perspective

The phosphatidylinositol 3′-kinase (PI3K)-protein kinase B (PI3K/AKT) signaling pathway serves as a crucial hub, activated by diverse cellular stimuli or injuries, governing fundamental cellular functions including transcription, translation, proliferation, growth, and survival. As shown in Table 1, growth factors binding to their receptor tyrosine kinase receptors initiate this pathway, with PI3K activation leading to AKT phosphorylation. Once activated, AKT orchestrates key cellular processes, modulating apoptosis, protein synthesis, metabolism, and the cell cycle.^67^^,^^68^

In the realm of cancer metastasis and epithelial–mesenchymal transition, the transcription factor RUNX1 (Runt-related transcription factor 1) emerges as a significant player, particularly in colorectal cancer. RUNX1's role in promoting colorectal cancer metastasis and regulating TGF-βⅠ-induced epithelial–mesenchymal transition is well-established.^69^^,^^70^ On a different front, miR-30b, abundantly released and accumulated in brown adipose tissue, takes the spotlight as a key regulator of brown adipose tissue function and adipocyte differentiation.^71^^,^^72^ Notably, miR-30b assumes an anti-fibrotic role by inhibiting adipose tissue fibrosis.

Recent research by Zhang et al^73^ illuminates the intricate interplay between miR-30b, RUNX1, and the PI3K/AKT pathway. Extracted from brown adipose tissue, miR-30b intervenes in epithelial–mesenchymal transition by directly targeting renal Runx1, curbing fibrosis. As shown in Table 1, by inhibiting RUNX1, miR-30b down-regulates PI3K, hindering AKT activation, and consequently mitigating adipose tissue fibrosis. This multifaceted regulatory mechanism underscores miR-30b′s pivotal role in modulating adipose tissue fibrosis and its potential implications in therapeutic interventions (Fig. 3).

## Balancing act: PPAR-γ and miR-155 in adipose tissue fibrosis regulation

NF-κB, a dimeric transcription factor, intricately orchestrates genes pivotal in immunity, inflammation, and cell survival.^74^ Based on previous discussions, cellular stimulation by tumor necrosis factor-α and other factors triggers the activation of NF-κB. Upon activation, NF-κB translocates to the nucleus,^75^ where it facilitates the transcription of miR-155.^76^ miR-155, in turn, plays a critical role in adipogenesis regulation by targeting peroxisome proliferator-activated receptor gamma (PPAR-γ), a nuclear receptor vital for adipogenic control.^77^

The delicate balance in PPAR-γ expression is a linchpin in adipogenesis. When miR-155 dampens PPAR-γ expression, adipogenesis stalls, causing adipogenic cells to transform into fibroblasts. This transformation fuels the accumulation of non-adipogenic cells, exacerbating adipose tissue fibrosis.^77^

Intriguingly, Kandy et al uncovered a pivotal connection in a high-fat diet-induced obese mouse model. As shown in Table 1 and Figure 3, miR-155, by targeting *PPARG*, actively curtails PPAR-γ expression, curbing adipogenesis and concurrently promoting adipose tissue fibrosis.^78^ This revelation unravels a crucial facet of the miR-155 puzzle, shedding light on its intricate role in the complex tapestry of adipose tissue fibrosis.

## Understanding the pathogenesis and therapeutic applications of miRNA in diverse adipose tissue fibrosis conditions

### miR-19a modulation of HIPK2: a novel therapeutic approach to inhibit corneal fibrosis

Homeodomain-interacting protein kinase 2 (HIPK2) is a pivotal serine/threonine kinase^79^ and a significant gene implicated in promoting renal fibrosis.^80^ HIPK2 exerts its influence by directly interacting with Smads, including Smad1, Smad2, and Smad3, thereby regulating Smad-dependent gene expression. As shown in Figure 3, HIPK2 emerges as a vital orchestrator of the TGF-β/Smad signaling pathway.^22^^,^^80^

In a study conducted by Shen et al,^22^ it was discovered that in ADSCs overexpressing miR-19a, the miR-19a secretome could be transmitted to corneal keratinocytes. This transmission led to the binding of miR-19a to its target gene *HIPK2*, resulting in the down-regulation of HIPK2 expression, as shown in Table 1. Consequently, TGF-β/Smad signaling was inhibited, leading to reduced expression of fibrosis markers such as collagen III, matrix metalloproteinase 9, fibronectin, and alpha-smooth muscle actin, along with other ECM components. This down-regulation significantly diminished cell viability and ECM degradation, impeding corneal keratinocyte differentiation and, consequently, inhibiting corneal fibrosis.

### RAS-MAPK signaling: miRNA-mediated regulation in fibrosis-associated pathways

**Fig. 4:**
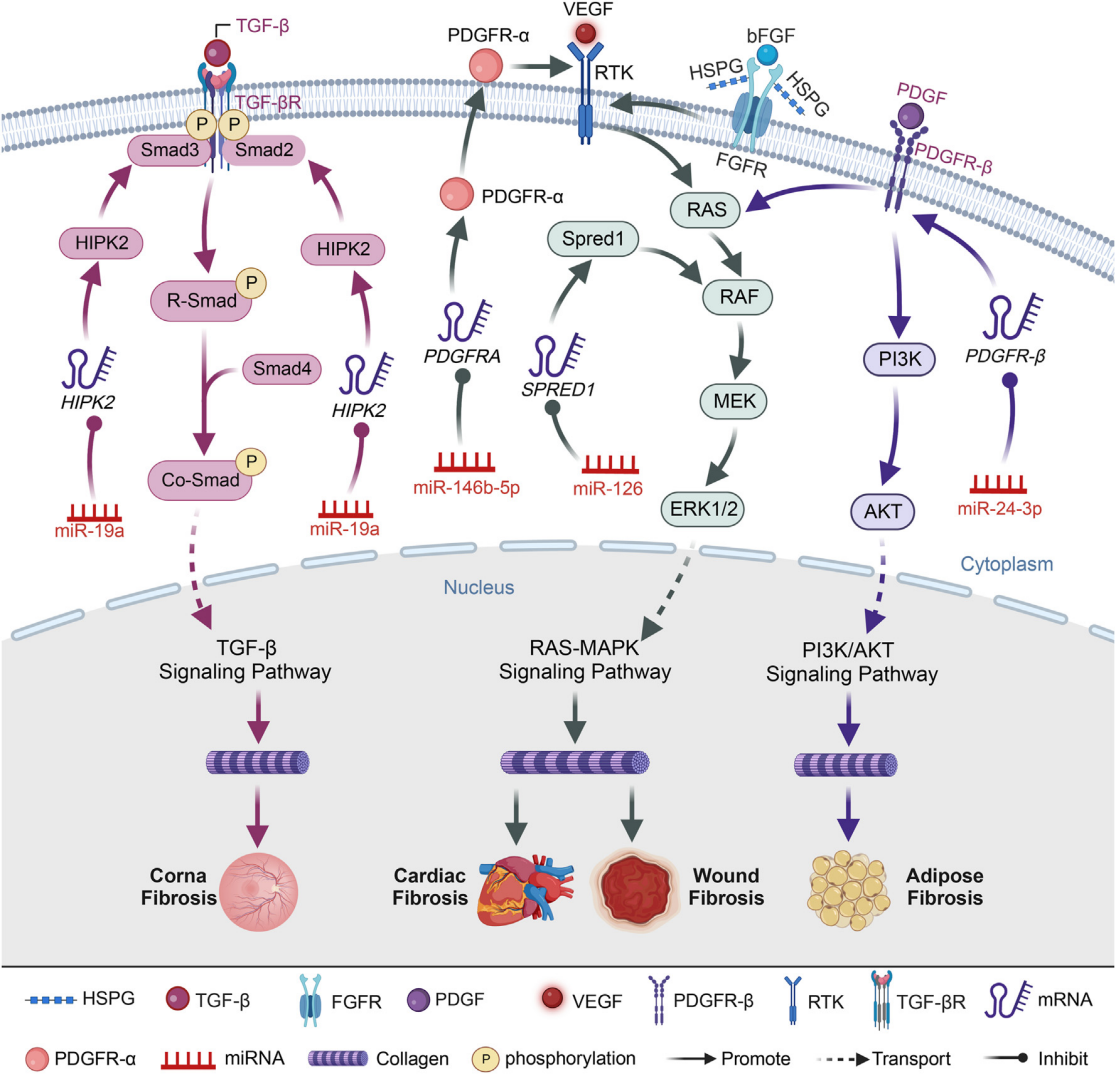
The etiology and utilization of miRNAs in different forms of adipose tissue fibrosis. Homeodomain interacting protein kinase 2 (HIPK2) plays a pivotal role in modulating the TGF-β/Smad3 signaling pathway. miR-19a directly interacts with HIPK2, down-regulating its expression and thereby inhibiting TGF-β/Smad signaling, effectively curbing corneal fibrosis. Decreased levels of *SPRED1* and *PI3KR2* result in the activation of VEGF signaling, enhancing the binding of VEGF (vascular endothelial growth factor) to receptor tyrosine kinase (RTK) on vascular endothelial cells. This activation leads to elevated PI3K (phosphoinositide 3 kinase) expression and subsequent activation of the PI3K/AKT pathway, resulting in increased expression of profibrotic proteins such as alpha-smooth muscle actin (α-SMA) and collagen, ultimately promoting tissue fibrosis. miR-126 targets *SPRED1* and *PI3KR2* in the heart, reducing the expression of Spred1 and PI3KR2. This reduction activates the VEGF signaling pathway and its downstream PI3K/AKT signaling cascade, fostering cardiac fibrosis. The binding of extracellular basic fibroblast growth factor (bFGF) to its receptor FGFR (fibroblast growth factor receptor) forms a dimer, which then associates with heparin sulfate proteoglycan (HSPG) on the cell surface, enhancing intracellular RTK activity. RTK recruits intracellular platelet-derived growth factor receptor alpha (PDGFR-α), activating the MAPK signaling pathway and influencing tissue fibrosis. miR-146b-5p targets *PDGFRA*, lowering PDGFR-α expression and restraining wound fibrosis. Platelet-derived growth factor receptor beta (PDGFR-β), upon binding with platelet-derived growth factor (PDGF), triggers the activation of the PI3K-AKT and RAS-MAPK pathways. This activation promotes collagen synthesis and deposition, escalating fibrosis. miR-24–3p targets *PDGFRB*, inhibiting PDGFR-β expression and dampening the activation of PI3K-AKT and RAS-MAPK signaling pathways, ultimately mitigating adipose tissue fibrosis. AKT, protein kinase B; MAPK, mitogen-activated protein kinase; PI3KR2, phosphoinositide 3-kinase regulatory subunit 2; RAS, Ras protein; Smad, mothers against decapentaplegic homolog; TGF-β, transforming growth factor β.

Sprouty-related EVH1 domain-containing protein 1 (SPRED1) plays a pivotal role in modulating vascular endothelial growth factor (VEGF) signaling.^81^ As shown in Figure 4, VEGF activates receptor tyrosine kinase, triggering the Ras protein (RAS) and subsequently activating Raf oncogene (RAF), leading to downstream MAP kinase-ERK kinase and extracellular signal-regulated kinases (ERK1/2) activation. This cascade enhances the RAS-MAPK signaling pathway,^82^ elevating collagen levels and promoting tissue fibrosis.^83^ Basic fibroblast growth factor is a multifunctional protein influencing cell growth, differentiation, and regeneration. Through receptor dimerization with fibroblast growth factor receptor and interaction with heparin sulfate proteoglycan, extracellular basic fibroblast growth factor heightens intracellular receptor tyrosine kinase activity.^84^ Receptor tyrosine kinase then recruits intracellular platelet-derived growth factor-α (PDGFR-α), activating the RAS-MAPK signaling pathway.^85^ PDGFR-α, a fibrosis-promoting receptor in various organs,^86^^,^^87^ is targeted by miR-146b-5p in wound granulation tissue fibroblasts.^85^

Furthermore, as shown in Table 1, miR-126, prevalent in vascular endothelial cells and vascular smooth muscle cells, significantly regulates physiological angiogenesis.^88^^,^^89^ Notably, its expression diminishes in fibrotic hearts.^90^ Luo et al^81^ demonstrated that ADSCs overexpressing miR-126 yield a miR-126-rich secretome. This secretome, delivered to cardiomyocytes, inhibits the expression of *SPRED1*, diminishing Spred1 levels in the heart. Consequently, it inhibits RAF phosphorylation, VEGF signaling, and cardiac fibrosis. Fujisawa et al^85^ revealed that injections of basic fibroblast growth factor augment miR-146b-5p levels in adipose tissue cells, reinforcing its binding to *PDGFRA*. This action diminishes PDGFR-α expression in fibrogenic progenitor cells, preventing profibrotic factor activation, myofibroblast formation, and wound fibrosis. These findings underscore the intricate regulatory mechanisms governing fibrosis-associated pathways.

### Fibrosis regulation: decoding the PDGFR-β signaling nexus and miRNA-mediated interventions

Platelet-derived growth factor receptor-β (PDGFR-β) is a pivotal receptor involved in liver fibrosis as well as fibrosis in multiple organs. As shown in Figure 4, PDGFR-β activation initiates two critical signaling pathways, PI3K-AKT and RAS-MAPK.^91^ Upon stimulation, HSCs display increased surface expression of PDGFR-β, leading to complex formation with platelet-derived growth factor. This complex activates both PI3K-AKT, promoting collagen synthesis, and RAS-MAPK, inhibiting tissue inhibitor of metalloproteinase 1 activity, hindering matrix degradation, and worsening fibrosis.^92^^,^^93^

Recent research by Cai et al^91^ unveiled a novel mechanism in adipose tissue fibrosis regulation. TGF-β down-regulates miR-24-3p expression in ADSCs. Human umbilical vein endothelial cells secrete TGF-β, which is captured by ADSCs, suppressing miR-24-3p expression. As shown in Table 1, miR-24-3p targets and inhibits PDGFR-β,^94^ thereby disrupting the activation of both PI3K-AKT and RAS-MAPK signaling pathways. This intricate regulatory network highlights the potential therapeutic implications for mitigating organ fibrosis.

## Discussion

### Unlocking potential: miRNA signatures as therapeutic targets in adipose tissue and organ fibrosis

Specific miRNA expression patterns have emerged as diagnostic markers for adipose tissue fibrosis, aiding early detection and monitoring of this condition. Studies have revealed elevated levels of liver-enriched miR-122 in mouse plasma after toxic acetaminophen exposure.^95^ Increased miR-122 expression has also been associated with liver damage and fatty liver conditions, both of which can lead to liver fibrosis.^96^, ^97^, ^98^, ^99^ miR-122 has been shown to influence the fibrotic process by regulating multiple signaling pathways, making it a potential therapeutic target for hepatic fatty fibrosis.^48^^,^^54^^,^^100^

Additionally, research by Wolfson et al^58^ demonstrated that down-regulation of miRNA-140 induced by a high-fat diet promotes mammary myofibroblast differentiation. Obesity-mediated TGF-β/Smad signaling down-regulates miR-140 expression, leading to breast myofibroblast differentiation and adipose tissue fibrosis. Conversely, miR-140 can mediate TGF-β/Smad signaling through negative feedback, aggravating adipose tissue fibrosis. Targeting and regulating miR-140 expression in breast cells via TGF-β/Smad signaling could provide a potential therapeutic approach for breast adipose tissue fibrosis.

In a diabetic mouse model, miR-30b has been shown to improve renal fibrosis response by inhibiting renal Runx1 and Snail1 expression.^73^ Massive release of miR-30b from brown adipose tissue and its accumulation in brown adipose tissue suggest the potential to regulate renal fibrosis by modulating miR-30b levels in brown adipose tissue. Consequently, miR-30b in adipose tissue stands as a promising target for treating diabetes-induced renal fibrosis.

Furthermore, miR-410-5p, exclusively expressed in kidney and adipose tissue, has been identified as an endocrine regulator promoting cardiac remodeling in metabolic disorders.^101^ Up-regulated miR-410–5p in the kidneys and fat of obese rats down-regulates its target protein Smad7 in the heart, activating the TGF-β signaling pathway and inducing fibrotic gene expression, thereby promoting cardiac fibrosis.^65^ miR-410-5p presents itself as a potential therapeutic target for cardiac fibrosis treatment. These discoveries underscore the potential of miRNA-based interventions in addressing fibrotic conditions across various organs and tissues.

### Fighting fibrosis: the role of miRNAs in tissue healing

Adipose tissue serves as a primary reservoir for miRNA, which plays a crucial role in modulating gene expression across distant organs.^73^ These miRNAs demonstrate potent anti-fibrotic effects, effectively impeding disease progression. Specifically, miR-30b derived from brown adipose tissue intervenes in epithelial–mesenchymal transition^102^ by directly targeting renal Runx1 expression, thereby exerting an anti-fibrotic influence. Notably, studies indicate that miR-155 deficiency suppresses TGF-β signaling, consequently mitigating steatosis and fibrosis in NASH.^39^ Furthermore, miR-155 down-regulates PPAR-γ expression, fostering adipose tissue fibrosis through *PPARG* targeting.^78^ Similarly, miR-146b-5p impedes fibroblast-to-myofibroblast transformation, diminishing cardiac fibrosis and facilitating cardiac remodeling after myocardial infarction.^103^ Additionally, miR-146b-5p hampers PDGFR-α expression in adipose tissue's fibrogenic progenitor cells, thus thwarting wound fibrosis.^85^ In summary, leveraging the distinctive miRNA expression profiles in various organs holds promise for combatting adipose tissue fibrosis effectively.

In sum, our investigation elucidates the mechanisms underlying miRNA's regulation of adipose and liver fibrosis, underscoring the significance of leveraging ADSC-secretome for miRNA delivery to distant targets. We aspire that our findings will inspire future researchers to delve into clinical investigations and applications of miRNA in combating adipose tissue fibrosis.

### Investigating miRNA-mediated approaches to combat liver fibrosis through ADSC secretome

Given the uncertainties surrounding the stability of transplanted stem cells, researchers are increasingly turning to miRNA transfection in ADSCs to explore the potential of their secretome, particularly in liver fibrosis studies. HSCs play a significant role in liver fibrosis, secreting collagen in response to liver injury, which can progress to cirrhosis.^47^^,^^104^ To slow down liver cirrhosis progression, inhibiting collagen expression and fibrosis-related genes has become a vital focus. Numerous studies have demonstrated the substantial therapeutic potential of the secretome in liver fibrosis treatment. Directly delivering miRNA to carbon tetrachloride-induced liver fibrosis models poses challenges. However, transfecting miRNA into ADSCs offers a viable solution, enabling effective delivery of miRNA to the target cells, exerting effects similar to endogenous miRNA,^24^^,^^25^ and sometimes yielding more robust anti-fibrotic results.^24^

For instance, Qu et al^25^ harnessed the secretome of miR-181-5p released from ADSCs overexpressing miR-181-5p to convey miR-181-5p into fibrotic stem cells, effectively mirroring the impact of endogenous miR-181-5p. Additionally, Paik et al^43^ discovered that the miR-150 secretome in ADSCs with miR-150 overexpression exerted potent anti-hepatic fibrosis effects and reduced systemic inflammatory responses both *in vivo* and *in vitro*. Lou et al^54^ elucidated that the ADSC-derived secretome mediates anti-hepatic fibrosis effects by modulating the miR-122 signaling pathway between ADSCs and HSCs. Furthermore, Park et al^24^ demonstrated that the miR-214 secretome in ADSCs overexpressing miR-214 displayed notably enhanced anti-fibrotic effects. Additionally, Shen et al^22^ revealed that ADSCs overexpressing miR-19a released the miR-19 secretome, delivering miR-19a to corneal keratinocytes and inhibiting TGF-β/Smad transmission, thereby preventing corneal fibrosis. These innovative approaches illuminate the promising potential of utilizing miR-modified ADSC secretome to combat liver fibrosis and other fibrotic conditions effectively.

### The potential application value of microRNA transfection of ADSCs in other disease fields

ADSCs engineered to overexpress specific miRNAs can efficiently deliver these molecules to target sites within organs, circumventing host immune rejection and exhibiting enhanced functionality. miR-342–5p, associated with atherosclerosis, when overexpressed in ADSCs, binds to protein phosphatase 1 regulatory subunit 12B in human atherosclerotic plaque models, fostering apoptosis of damaged human umbilical vein endothelial cells and thereby safeguarding against atherosclerosis.^105^ Similarly, miR-378, pivotal in cell proliferation, angiogenesis, and metastasis, targets suppressor of Fused (Sufu).^106^ Transfection of ADSCs with miR-378 results in its overexpression, down-regulating Sufu expression, activating the Sonic Hedgehog pathway, bolstering neovascularization and osteogenesis, and mitigating glucocorticoid-induced femoral head necrosis.^107^ miR-31, down-regulated in the middle cerebral artery occlusion model, mitigates neuronal damage, brain infarct volume, and oxidative stress in ischemic stroke mice when overexpressed.^108^ ADSCs overexpressing miR-31 markedly inhibit expression of tumor necrosis factor receptor associated factor 6, consequently reducing expression of downstream interferon regulatory factor 5, and enhancing neuroprotection against ischemic brain injury in mice.^109^ Moreover, miR-22, down-regulated in Alzheimer's disease, alleviates Alzheimer's disease-associated inflammation by targeting gasdermin D and inhibiting pyroptosis.^110^ ADSCs overexpressing miR-22 outperform miR-22 alone in inhibiting gasdermin D expression and enhancing cognitive function in Alzheimer's disease mice.^111^ Notably, in immune thrombocytopenia patients, miR-199a-5p levels are initially low but increase post-treatment.^112^ ADSCs overexpressing miR-199a-5p effectively attenuate immune thrombocytopenia progression.^113^ In summary, ADSCs engineered to overexpress miRNAs exhibit significant therapeutic efficacy across various diseases, holding promise for diverse clinical applications in disease treatment.

## Conclusions

The miRNA synthesized in adipose tissue plays a dual role by engaging in its own fibrosis process while also inhibiting adipose tissue fibrosis. Administration of exogenous miRNA augments its capacity to counteract adipose tissue fibrosis and impede its progression. By engineering an ADSC-secretome that overexpresses miRNA, anti-fibrotic miRNA can be transported to the liver, amplifying its miRNA expression and effectively thwarting fibrosis development. Moreover, ADSCs overexpressing miRNA hold promise for delivering miRNA to various target organs for the treatment of diverse conditions, including atherosclerosis, femoral head necrosis, ischemic stroke, neurodegenerative diseases, and autoimmune disorders. Nevertheless, our study faces certain limitations. Firstly, due to the scarcity of research on miRNA's role in adipose tissue fibrosis, our discussion centers on an overview of fibrosis in ADSCs overexpressing miRNA. Secondly, our search in public databases failed to yield clinical studies on miRNA's efficacy in treating adipose tissue fibrosis.

In sum, our investigation elucidates the mechanisms underlying miRNA's regulation of adipose and liver fibrosis, underscoring the significance of leveraging ADSC-secretome for miRNA delivery to distant targets. We aspire that our findings will inspire future researchers to delve into clinical investigations and applications of miRNA in combating adipose tissue fibrosis.

## Author contributions

M.T., Y.Z., Y.G., Q.X., X.Z., J.S., J.G., L.W., and S.D. were responsible for the collection and analysis of literature, as well as the preparation of figures and manuscript writing. M.T., Q.X., X.Z., J.S., and J.G. were involved in the collection literature and manuscript writing. M.T., Y.Z., and Y.G. were responsible for drawing pictures. S.D. and L.W. provided final approval for the submitted version of the manuscript. All authors reviewed and agreed to the final published version of the manuscript.

## Funding

This study is supported in part by the 10.13039/501100004731Zhejiang Provincial Natural Science Foundation of China (No. LTGD24H070006, LTGD23H150001), the Medical and Health Research Project of Zhejiang Province, China (No. 2023KY212, 2024KY631), and the Zhejiang Province Administration of Traditional Chinese Medicine (China) (No. 2024ZL249).

## Declaration of competing interest

The authors declared no competing interests.
